# Three-year follow-up of de-escalated axillary treatment after neoadjuvant systemic therapy in clinically node-positive breast cancer: the MARI-protocol

**DOI:** 10.1007/s10549-022-06545-z

**Published:** 2022-03-03

**Authors:** Ariane A. van Loevezijn, Marieke E. M. van der Noordaa, Marcel P. M. Stokkel, Erik D. van Werkhoven, Emma J. Groen, Claudette E. Loo, Paula H. M. Elkhuizen, Gabe S. Sonke, Nicola S. Russell, Frederieke H. van Duijnhoven, Marie-Jeanne T. F. D. Vrancken Peeters

**Affiliations:** 1 Department of Surgical Oncology, Netherlands Cancer Institute - Antoni Van Leeuwenhoek, Amsterdam, The Netherlands; 2 Department of Nuclear Medicine, Netherlands Cancer Institute - Antoni Van Leeuwenhoek, Amsterdam, The Netherlands; 3 Department of Biometrics, Netherlands Cancer Institute - Antoni Van Leeuwenhoek, Amsterdam, The Netherlands; 4 Department of Pathology, Netherlands Cancer Institute - Antoni Van Leeuwenhoek, Amsterdam, The Netherlands; 5 Department of Radiology, Netherlands Cancer Institute - Antoni Van Leeuwenhoek, Amsterdam, The Netherlands; 6 Department of Radiation Oncology, Netherlands Cancer Institute - Antoni Van Leeuwenhoek, Amsterdam, The Netherlands; 7 Department of Medical Oncology, Netherlands Cancer Institute - Antoni Van Leeuwenhoek, Amsterdam, The Netherlands; 8 Department of Surgery, Amsterdam University Medical Center, Meibergdreef 9, 1105 AZ Amsterdam, The Netherlands

**Keywords:** Breast cancer, Axillary lymph node dissection, Neoadjuvant therapy, Tailored treatment

## Abstract

**Purpose:**

In clinically node-positive (cN+) breast cancer patients, evidence supporting response-guided treatment after neoadjuvant systemic therapy (NST) instead of axillary lymph node dissection (ALND) is increasing, but follow-up results are lacking. We assessed three-year axillary recurrence-free interval (aRFI) in cN+ patients with response-adjusted axillary treatment according to the ‘Marking Axillary lymph nodes with Radioactive Iodine seeds’ (MARI)-protocol.

**Methods:**

We retrospectively assessed all stage II–III cytologically proven cN+ breast cancer patients who underwent the MARI-protocol between July 2014 and November 2018. Pre-NST axillary staging with FDG-PET/CT (less- or more than four suspicious axillary nodes; cALN < 4 or cALN ≥ 4) and post-NST pathological axillary response measured in the pre-NST largest tumor-positive axillary lymph node marked with an iodine seed (MARI-node; ypMARI-neg or ypMARI-pos) determined axillary treatment: no further treatment (cALN < 4, ypMARI-neg), axillary radiotherapy (ART) (cALN < 4, ypMARI-pos and cALN ≥ 4, ypMARI-neg) or ALND plus ART (cALN ≥ 4, ypMARI-pos).

**Results:**

Of 272 women included, the MARI-node was tumor-negative in 56 (32%) of 174 cALN < 4 patients and 43 (44%) of 98 cALN ≥ 4 patients. According to protocol, 56 (21%) patients received no further axillary treatment, 161 (59%) received ART and 55 (20%) received ALND plus ART. Median follow-up was 3.0 years (IQR 1.9–4.1). Five patients (one no further treatment, four ART) had axillary metastases. Three-year aRFI was 98% (95% CI 96–100). The overall recurrence risk remained highest for patients with ALND (HR 4.36; 95% CI 0.95–20.04, *p* = 0.059).

**Conclusions:**

De-escalation of axillary treatment according to the MARI-protocol prevented ALND in 80% of cN+ patients with an excellent three-year aRFI of 98%.

## Introduction

In clinically node-positive (cN+) breast cancer patients, axillary lymph node dissection (ALND) is still widely considered the standard of care [1–3]. The ongoing shift from adjuvant to neoadjuvant systemic therapy (NST) however, allows consideration of less extensive axillary surgery for cN+ patients [4, 5]. Currently, a pathologic complete response (pCR) of the axilla (ypN0) is seen in one-third of cN+ patients with NST, with pCR rates of more than 50% in triple-negative and HER2-positive patients [6]. Patients with axillary pCR are unlikely to benefit from ALND, while facing surgical complications and long-term morbidity such as lymphedema and limitation of shoulder motion. Therefore, strategies to de-escalate axillary treatment in cN+ patients are being investigated [7–9].

At the Netherlands Cancer Institute, the Marking Axillary Lymph Nodes with Radioactive Iodine seeds (MARI)-procedure [10] was developed to re-stage the axilla after NST. The largest tumor-positive axillary lymph node (ALN) was marked with an iodine seed pre-NST (MARI-node) and selectively removed and assessed post-NST [11]. This procedure was found to be a reliable measurement of axillary response with a false-negative rate of only 7% [10–12]. Hereafter, an axillary treatment algorithm was developed (i.e., MARI-protocol) which combined the outcome of the MARI-procedure (ypMARI-neg or ypMARI-pos) with a pre-NST acquired fluorodeoxyglucose (FDG)-positron emission tomography/computed tomography (PET/CT) scan to determine the presence of less or more than four (cALN < 4 or cALN ≥ 4) tumor-positive ALNs prior to NST [11, 12]. Patients staged cALN < 4, ypMARI-neg received no further axillary treatment, patients staged cALN < 4, ypMARI-pos and cALN ≥ 4, ypMARI-neg received axillary radiotherapy (ART) and patients staged cALN ≥ 4, ypMARI-pos received ALND plus ART [12].

Long-term outcomes of patients treated according to the MARI-protocol have not yet been reported. In this study, we assessed three-year follow-up results and in particular axillary recurrence-free interval (aRFI) of clinically node-positive breast cancer patients who underwent tailored and de-escalated axillary treatment after NST according to the MARI-protocol.

## Methods

### Patient selection

This is a single-center cohort study including prospectively registered patients. We included all women, 18 years or older, with stage II–III pathologically proven axillary cN+ breast cancer of any subtype, who underwent the MARI-protocol between July 2014 and November 2018 at the Netherlands Cancer Institute. Exclusion criteria were history of breast cancer and non-FDG-avid breast cancer. This study was approved by the institutional review board of the Netherlands Cancer Institute.

### Diagnostic procedures

Core needle biopsies of the breast tumor were obtained to determine histological subtype, hormone receptor and HER2- status. Hormone receptor status was defined as positive if estrogen expression was ≥ 10%, and HER2-status was regarded positive if 3 + or 2 + with positive in-situ hybridization, according to ASCO-CAP guidelines [13]. Tumor grade was determined according to the modified Bloom-Richardson method [14]. The size and extent of the primary tumor were assessed by mammography, ultrasound and dynamic contrast-enhanced (DCE) MRI. All patients underwent axillary and peri-clavicular ultrasound. Ultrasound-guided fine needle aspiration (FNA) was performed in case of suspect lymph nodes.

A whole body FDG-PET/CT (Philips Gemini, Cleveland, OH, USA) was performed for regional staging and detection of distant metastasis. PET acquisition was followed by a low-dose CT scan (40 mAs, 2 mm slices). Additional PET/CT images in prone position were acquired if patients were scanned at the Netherlands Cancer Institute. The uptake of FDG-positive ALNs was assessed by experienced nuclear medicine physicians and was discussed during multidisciplinary consultations. A lymph node was regarded as highly suspicious for metastasis when the uptake was higher than the blood pool activity. For axillary staging according to the MARI-protocol, the number of FDG-positive ALNs was used rather than the clinical TNM classification. Patients with less than four FDG-positive axillary nodes on PET/CT were defined as cALN < 4 and patients with more than three FDG-positive axillary nodes were defined as cALN ≥ 4, regardless of presence of peri-clavicular or internal mammary chain nodes.

### Radioactive seed localization

In all patients, an Iodine seed (STM1251, Bard Brachytherapy Inc., Carol Stream, IL) with an apparent activity varying from 0.2 to 1.0 MBq at time of implementation was placed under ultrasound guidance in the largest pathology proven tumor-positive axillary lymph node (i.e., MARI-node) prior to the start of the first NST cycle. The activity of Iodine seeds used for MARI-node localization is lower than for breast tumor localization (apparent activity 1.0–7.6 Mbq) [15, 16] to minimize irradiation of the node. Marking of the breast tumor was performed during the same procedure. Adequate position of the markers in the breast and axilla was confirmed by ultrasound and/or mammography. A comprehensive description of the MARI-procedure and radiation safety protocols has been described previously [17].

### Treatment and response evaluation

Neoadjuvant systemic therapy was administered according to institutional guidelines as previously described [11]. After completion of NST, surgery of the breast and selective removal of the MARI-node was performed. A gamma probe was used to guide the localization of the Iodine seeds and surgical resection. Additional axillary nodes were removed when a lymph node was located directly adjacent to the MARI-node.

In cALN < 4 patients, the MARI-node was formalin-fixed overnight followed by hematoxylin and eosin (H&E) and cytokeratin staining at a single level. An intraoperative frozen section of the MARI-node was obtained in all cALN ≥ 4 patients. For intraoperative frozen sections, 2 mm tissue slices ware made from which 5 µm H&E sections were prepared and assessed. Hereafter, the tissue was also fixed in formalin overnight followed by a new H&E and a cytokeratin stain at a single level.

Pathological complete response of the axilla was defined as the absence of vital tumor cells in the removed axillary lymph node(s) (ypN0). A pCR of the breast was defined as absence of invasive and in-situ carcinoma in the breast (ypT0).

### Tailored and de-escalated axillary treatment

All cALN < 4 patients with pCR of the MARI-node (ypMARI-neg) received no further axillary treatment. Axillary levels I to IV were irradiated in patients staged cALN < 4, ypMARI-pos and cALN ≥ 4, ypMARI-neg. ALND and ART was performed in all patients staged cALN ≥ 4, ypMARI-pos. The ALND was performed in a second operation in patients with a false-negative intraoperative frozen section of the MARI-node.

Patients with ART underwent irradiation to the axillary and infra/supraclavicular nodes, and in case of FDG-positive nodes in the internal mammary chain (IMC), the IMC was included. Delineation of lymph node levels was performed according to the Danish national delineation guidelines, and from January 2015, according to the European Society for Radiotherapy and Oncology consensus guidelines. A dose of 42.56 Gy in 16 fractions of 2.66 Gy was prescribed, or 46.2 Gy in 21 fractions of 2.2 Gy if a simultaneous boost dose was given to the tumor bed in the breast. The radiotherapy technique used was either static field Intensity Modulated RadioTherapy (IMRT) or Volumetic Modulated Arc Therapy (VMAT) planning. Deep Inspiration Breath Hold Technique was applied for all left sided breast tumors.

Patients received adjuvant systemic treatment according to institutional guidelines. Patients with hormone-receptor positive tumors received adjuvant hormonal therapy and all patients with HER2-positive tumors received adjuvant HER2-directed therapy. Following the publication of the CREATE-X trial in 2017 [18], adjuvant Capecitabine was administered in all patients with triple-negative breast cancer with residual disease and a selection of estrogen receptor-positive tumors with residual disease.

### Outcomes

The primary endpoint was three-year axillary recurrence-free interval (aRFI), defined as tumor recurrence in lymph nodes in the ipsilateral axilla. Secondary outcomes were local-, regional-,distant and overall- RFI rates and overall survival. Axillary recurrence-free interval was defined as time from the MARI-procedure to axillary recurrence or death from any cause. Patients who died without axillary recurrence or were lost to follow-up were censored in the analysis. Patients who developed (and received treatment) for another event (e.g. local recurrence, distant metastases, or new primary) before axillary recurrence were censored in the analysis, except if it was a synchronous event (i.e., diagnosed at subsequent disease staging). In addition, three-year RFI was assessed in the pre-specified treatment groups (i.e., no further treatment [cALN < 4, ypMARI-neg] ART [cALN < 4, ypMARI-pos and cALN ≥ 4, ypMARI-neg] and ALND plus ART [cALN ≥ 4, ypMARI-pos], as well as factors influencing disease recurrence (i.e., age, clinical stage, subtype and pathological response) were evaluated.

### Statistical analysis

Recurrence-free interval and overall survival of the four treatment groups were estimated by the Kaplan–Meier method and compared with log-rank tests. All survival estimates were reported with their 95% confidence intervals. To evaluate associations between patient characteristics, axillary treatment and recurrence-free interval, Cox proportional-hazards models were used. The two-sided 95% confidence intervals for proportions were calculated using the exact Clopper-Pearson method. Baseline characteristics were compared between patients staged cALN < 4 and cALN ≥ 4 with an independent sample t test for sample means and with Pearson Chi-square or Fisher’s exact test for categorical variables. Statistical significance for comparisons between groups was defined as p < 0.05. All statistical analyses were performed in IBM SPSS Statistics, version 25.0.

## Results

### Patient characteristics

**Fig. 1:**
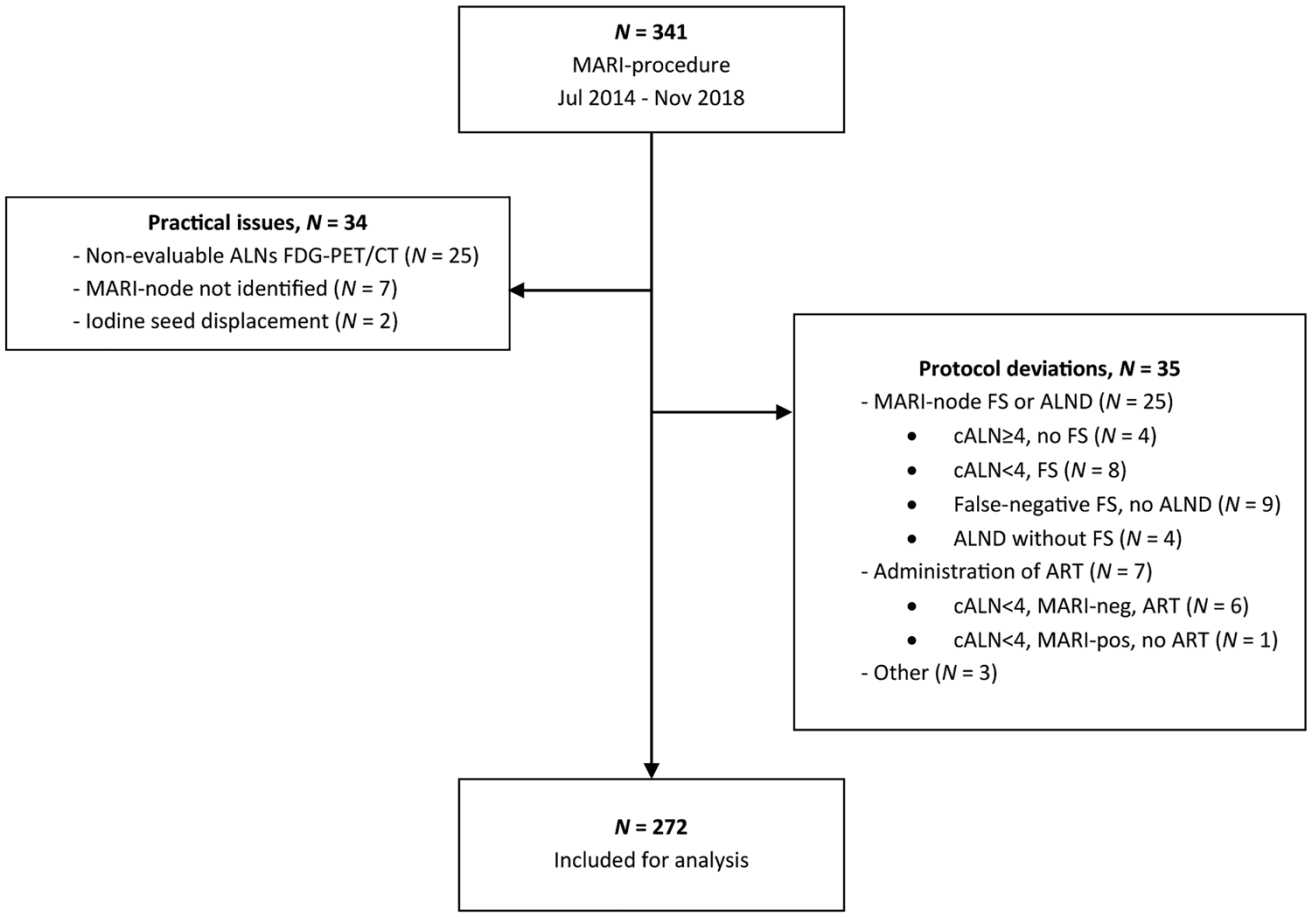
Patient inclusion. *MARI* Marked axillary lymph node with radioactive iodine seed, *FDG-PET/CT* fluorodeoxyglucose—positron emission tomography/computed tomography; *ALNs* Axillary lymph nodes, *FS* frozen section, *ALND* axillary lymph node dissection, *cALN* < *4* less than four FDG-PET/CT-positive axillary lymph nodes, *cALN* ≥ *4* more than four FDG-PET/CT positive axillary lymph nodes, *ART* axillary radiotherapy

Between July 2014 and November 2018, 272 (80%) of 341 prospectively registered patients who underwent the MARI-procedure fulfilled eligibility criteria (Fig. 1). Reasons for exclusion were practical issues (*N* = 34) (e.g. non-FDG avid or clustered, indistinguishable ALNs) or protocol deviations (*N* = 35) (e.g. false-negative intraoperative frozen section not followed by ALND).

Baseline characteristics are shown in Table 1. Median age was 48 years (range 22–79) and the majority of patients had invasive carcinoma of no special type (89%). Staging with FDG-PET/CT prior to NST categorized 174 (64%) patients as cALN < 4 and 98 (36%) patients as cALN ≥ 4. Baseline characteristics differed between the groups: more HER2-positive tumors (38% vs. 23%) and less HR-positive/HER2-negative tumors (43% vs. 57%) were found in cALN ≥ 4 patients compared to cALN < 4 patients (*p* = 0.012, Table 1).

**Table 1:** Baseline patient and tumor characteristics

	Total *N* = 272		cALN < 4 *N* = 174		cALN ≥ 4 *N* = 98		*P* value
Age (y)	48	(41–56)	48	(40–55)	49	(42–56)	0.981
Diagnostic imaging							
Tumor size MRI (mm)	32	(22–50)	31	(22–46)	36	(24–55)	0.109
PET/CT-positive ALNs	2	(1–4)	1	(1–2)	5	(4–7)^a^	< 0.001
Histology							0.797
No special type^a^	242	(89%)	153	(88%)	89	(91%)	
Lobular	29	(11%)	20	(11%)	9	(9%)	
Other	1	(1%)	1	(1%)	0	–	
Tumor subtype							0.012
HR + / HER2 −	140	(51%)	99	(57%)	41	(43%)	
HR + / HER2 +	46	(17%)	27	(15%)	19	(19%)	
HR − / HER2 +	32	(12%)	13	(8%)	19	(19%)	
Triple-negative	54	(20%)	35	(20%)	19	(19%)	
Bloom-Richardson grade							0.565
Grade 1	9	(4%)	7	(4%)	2	(2%)	
Grade 2	135	(53%)	90	(55%)	45	(51%)	
Grade 3	110	(43%)	68	(41%)	42	(47%)	
Unknown	18	–	9	–	9	–	

Data are median (IQR) or *N* (%)

*cALN* < *4* less than four FDG-PET/CT-positive axillary lymph nodes, *cALN* ≥ *4* more than four FDG-PET/CT positive axillary lymph nodes, *MARI* marked axillary lymph node with radioactive iodine seed, *ALNs* axillary lymph nodes, *ALND* axillary lymph node dissection

^a^The number of ALNs was reported as ‘multiple’ in 26 patients. +formerly known as invasive ductal carcinoma. All characteristics were assessed before administration of neoadjuvant systemic therapy

### The MARI-procedure

The total number of ALNs removed during the MARI-procedure ranged from one to six, with a median of one (IQR 1–2). A pCR of the MARI-node (ypMARI-neg) was found in 56 (32%) of 174 cALN < 4 patients and in 43 (44%) of 98 cALN ≥ 4 patients (*p* = 0.054) and varied per subtype, with rates of 9% (13 of 140) in HR-positive/HER2-negative tumors, 59% (27 of 46) in HR-positive/HER2-positive tumors, 94% (30 of 32) in HR-negative/HER2-positive tumors and 54% (29 of 54) in triple-negative tumors (*p* < 0.001). In all patients with a tumor-negative MARI-node, the additionally removed ALNs were negative as well.

Breast pCR occurred in 78 (29%; 95% CI 23–34) patients and 64 (24%; 95% CI 19–29) patients had both pCR of the breast and the MARI-node (ypT0N0).

### Tailored axillary treatment

**Fig. 2:**
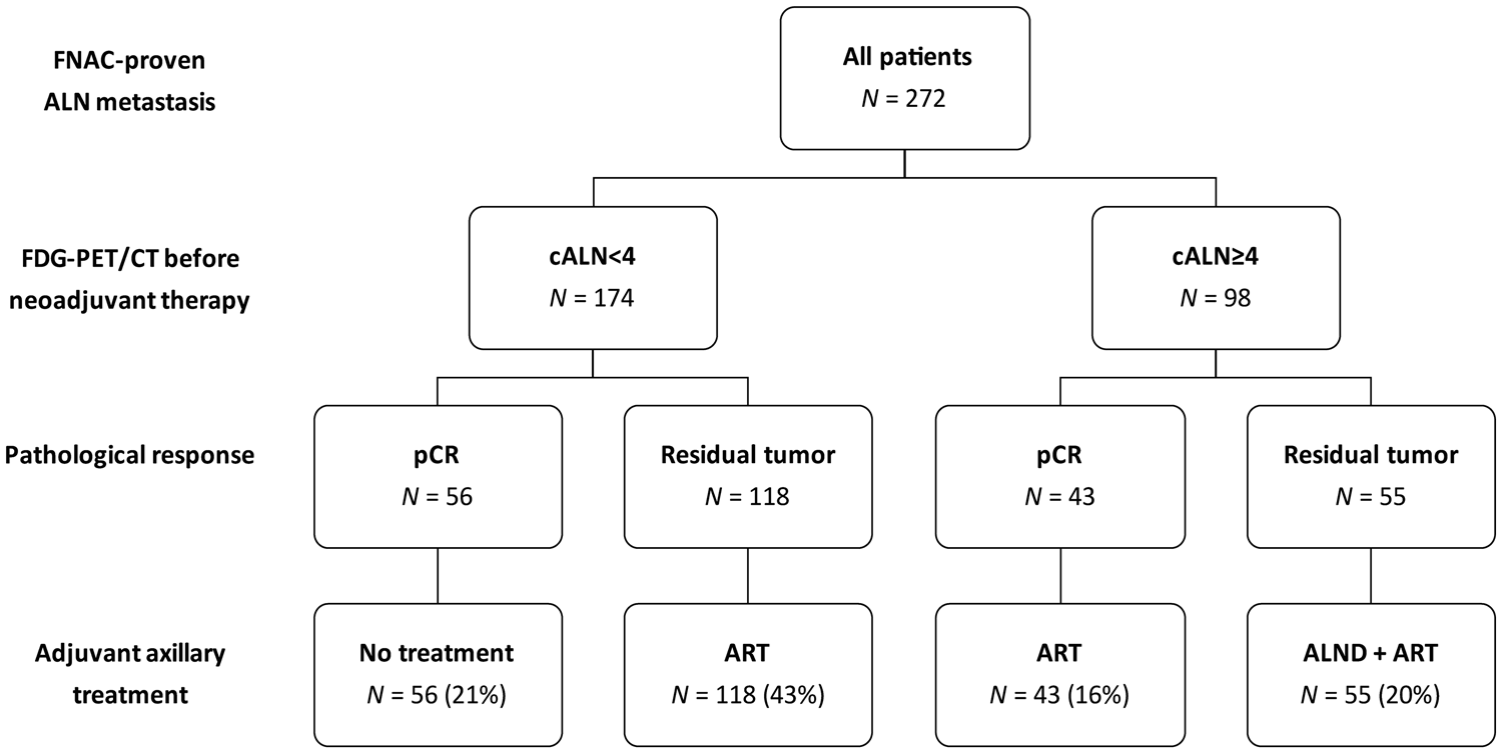
Tailored adjuvant axillary treatment strategy according to the MARI protocol. *FNAC* fine needle aspiration cytology, *cALN* < *4* less than four FDG-PET/CT-positive axillary lymph nodes, *cALN* ≥ *4* more than four FDG-PET/CT positive axillary lymph nodes, *MARI* marked axillary lymph node with radioactive iodine seed, *pCR* pathological complete response, *ALN* Axillary lymph node, *ALND* axillary lymph node dissection, *ART* axillary radiotherapy

Axillary treatment according to the MARI-protocol is presented in Fig. 2 and resulted in omission of ALND in a total of 217 (80%) patients: no further axillary treatment was administered in 56 (21%) patients (cALN < 4, ypMARI-neg), and 161 (59%) patients (118 cALN < 4, ypMARI-pos and 43 cALN ≥ 4, ypMARI-neg) received ART. Fifty-five (20%) cALN ≥ 4 patients had residual tumor in the MARI-node and underwent ALND plus ART. Adjuvant systemic therapy was administered in 228 (84%) patients and included chemotherapy in 44 (16%) patients, HER2-directed therapy in 80 (29%) patients and hormonal therapy in 183 (67%) patients.

### Axillary recurrence

Median follow-up was 3.0 years (IQR 1.9–4.1, range 0.3–5.4). Axillary recurrences occurred in a total of five (1.8%) patients, and three-year aRFI was 98% (95% CI 96–100). All five were cALN < 4 patients with synchronous other metastases. Subtype was triple-negative in four patients and HR-positive/HER2negative in one. One of the five patients had pCR of the MARI-node and therefore received no further axillary treatment. In this patient, extensive metastases were found in the axilla, lower neck and cervical region. The remaining four patients had residual disease in the MARI-node and underwent radiation treatment. Of these, one patient had axillary and IMC metastases, one patient had axillary metastases with concurrent metastases in the breast/thoracic wall, supraclavicular nodes and in the IMC, and two patients had axillary metastases with synchronous distant metastases.

### Secondary outcomes

In total, 27 (9.9%) patients developed one or more recurrences (distant, regional or local). Distant metastases were found in 19 (7.0%) patients, regional nodal recurrences (including the five patients with axillary metastases) occurred in 10 (3.7%) patients and a local recurrence was detected in 6 (2.2%) patients. The corresponding overall three-year RFI and distant, regional, and local RFI rates were 90% (95% CI 86–94), 93% (95% CI 90–96), 96% (95% CI 94–99) and 98% (95% CI 95–100), respectively. Sixteen (5.9%) patients died, al due to breast cancer recurrence, resulting in a three-year overall and breast cancer survival of 95% (95% CI 91–98).

**Fig. 3:**
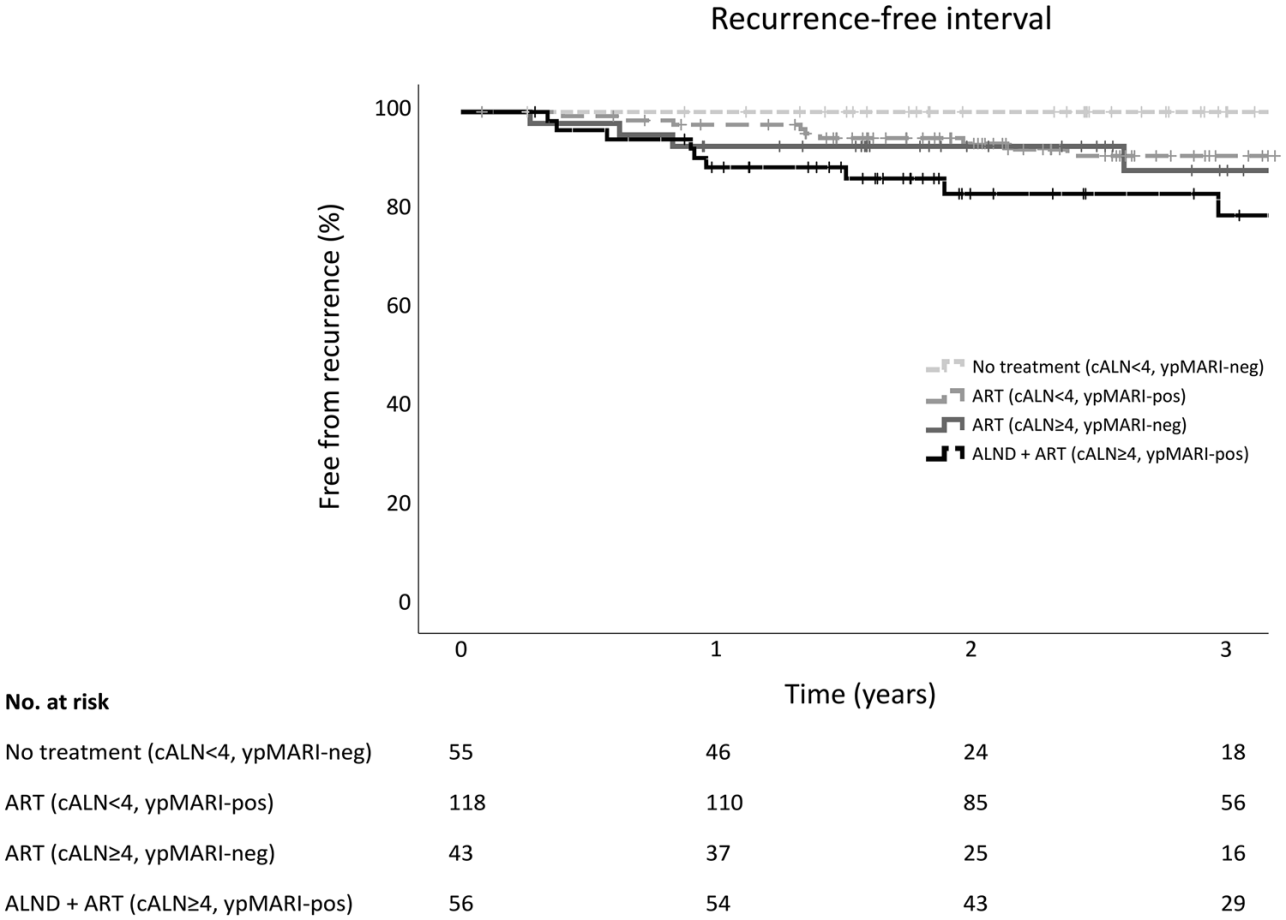
Overall recurrence-free interval by axillary staging and treatment. *cALN* < *4* less than four FDG-PET/CT-positive axillary lymph nodes, *cALN* ≥ *4* more than four FDG-PET/CT positive axillary lymph nodes, *MARI* marked axillary lymph node with radioactive iodine seed, *ypMARI-neg/ypMARI-pos* pathology analysis of MARI-node after neoadjuvant systemic therapy tumor-negative/tumor-positive, *ART* axillary radiotherapy, *ALND* axillary lymph node dissection

The first documented site(s) of recurrence by axillary treatment group are shown in Table 2. In total, fewest recurrences (5%) occurred in cALN < 4, ypMARI-neg patients with no further axillary treatment. Nine percent recurrences were found in both ART groups (cALN < 4 and cALN ≥ 4) and 18% in the ALND group (Table 2). The corresponding three-year RFI rates were 100% (95% CI n.a.), 91% (95% CI 85–97), 88% (95% CI 76–100) and 79% (95% CI 66–92) (Fig. 3). In an exploratory analysis, the trend in increased risk of disease recurrence for cALN ≥ 4, ypMARI-pos patients remained after adjusting for age, subtype and pathological response of the breast (HR 4.36, 95% CI 0.95–20.04, *p* = 0.059).

**Table 2:** Locations of breast cancer recurrence by response adjusted axillary treatment group

	cALN < 4		cALN ≥ 4		
	MARI pCR	MARI tumor +	MARI pCR	MARI tumor +	
	*No treatment*	*ART*	*ART*	*ALND* + *ART*	Total
	*N* = 56	*N* = 118	*N* = 43	*N* = 55	*N* = 272
Total patients with event per treatment group^a^					
Axillary + local	0	1	0	0	1
Axillary + regional	1	1	0	0	2
Axillary + distant	0	2	0	0	2
Local	1	0	0	2	3
Local + regional	0	0	0	1	1
Local + distant	0	0	1	0	1
Regional	0	0	0	1	1
Regional + distant	0	0	1	2	3
Distant	1	6	2	4	13
Total	3 (5.4%)	10 (8.5%)	4 (9.3%)	10 (18.2%)	27 (9.9%)
Total patients with event by location					
Axillary	1	4	0	0	5 (1.8%)
Local	1	1	1	3	6 (2.2%)
Regional (incl. axilla)	1	4	1	4	10 (3.7%)
Distant	1	8	4	6	19 (7.0%)

*cALN* < *4* less than four FDG-PET/CT-positive axillary lymph nodes, *cALN* ≥ *4* more than four FDG-PET/CT positive axillary lymph nodes, *MARI* marked axillary lymph node with radioactive iodine seed, *pCR* pathological complete response, *tumor* + tumor-positive, *ART* axillary radiotherapy, *ALND* axillary lymph node dissection

^a^Axillary recurrences were reported separately from non-axillary regional nodal metastases; Lower neck/cervical metastases were considered regional metastases

Baseline characteristics associated with increased risk of disease recurrence in univariate analysis were clinical stage cALN ≥ 4 (HR 2.25, 95% CI 1.05–4.79, *p* = 0.036) and triple-negative breast cancer (HR 2.89, 95% CI 1.23–6.81, *p* = 0.015) (Table 3). In multivariate analysis, triple-negative breast cancer (HR 4.32, 95% CI 1.74–10.53, *p* = 0.002) and residual tumor in the MARI-node (HR 3.13, 95% CI 1.02–9.68, *p* = 0.047) were significantly associated with disease recurrence.

**Table 3:** Cox regression analysis for overall recurrence-free interval

	Events		Univariate			Multivariate		
	*N*	*(%)*	HR	95% CI	*P* value	HR	95% CI	*P* value
Age, years	27	(10%)	1.01	0.98–1.05	0.517	1.01	0.97–1.05	0.582
Subtype								
HR + / HER2 −	10	(7%)	Ref					
HR + / HER2 +	3	(7%)	0.99	0.27–3.58	0.981	1.57	0.40–6.10	0.519
HR − / HER2 +	3	(9%)	1.33	0.37–4.84	0.666	3.39	0.63–18.12	0.154
Triple-negative	11	(20%)	2.89	1.23–6.81	0.015	4.28	1.74–10.53	0.002
Clinical tumor stage								
≤ cT1	2	(4%)	Ref					
cT2	16	(10%)	2.72	0.63–11.85	0.182	2.91	0.66–12.81	0.157
≥ cT3	9	(14%)	4.06	0.88–18.82	0.073	3.68	0.78–17.49	0.101
Clinical ALN group								
cALN < 4	13	(8%)	Ref					
cALN ≥ 4	14	(14%)	2.25	1.05–4.79	0.036	1.96	0.88–4.35	0.100
Pathology MARI node(s)								
Tumor-negative	7	(7%)	Ref					
Tumor-positive	20	(12%)	1.67	0.71–3.95	0.244	3.13	1.02–9.68	0.047
Pathology breast								
Residual disease	23	(12%)	Ref					
Complete response	4	(5%)	0.45	0.15–1.29	0.137			
Adjuvant axillary treatment^a^								
No further treatment	3	(5%)	Ref					
ART (cALN < 4)	10	(9%)	1.64	0.45–5.97	0.451			
ART (cALN ≥ 4)	4	(9%)	2.04	0.46–9.13	0.351			
ALND plus ART	10	(18%)	4.18	1.15–15.22	0.030			

*HR* hazard ratio; *cALN* < *4* less than four FDG-PET/CT-positive axillary lymph nodes; *cALN* ≥ *4* more than four FDG-PET/CT positive axillary lymph nodes, *MARI* marked axillary lymph node with radioactive iodine seed, *ART* axillary radiotherapy, *ALND* axillary lymph node dissection

^a^Adjuvant axillary treatment was not included in multivariate analysis due to collinearity with clinical axillary lymph node group and pathology MARI node(s) (*R*^2^ ≥ 0.6)

## Discussion

This study demonstrates that tailored de-escalated axillary treatment after NST according to the MARI-protocol in cN+ breast cancer patients is safe with an 80% reduction in ALNDs and excellent three-year aRFI and regional RFI of 98% and 96%, respectively. As axillary recurrences occur at a median of two years following treatment [19–21], the high aRFI of 98% we found at a median follow-up of three years can be considered a significant result.

Previously reported regional RFS rates in cN+ patients who underwent complete ALND after NST included rates of 96% at 3 years follow-up [22], 94–96% at years follow-up [23–27] and 91–95% at ten years follow-up [28]. Notably, the number of cN2-3 patients we included was generally higher (36% cALN ≥ 4 patients), and the high RFS we found is therefore less likely to result from a more favorable patient selection. Several studies have established the significance of clinical stage and especially pathological axillary response as prognostic factors [24, 28–31]. Accordingly, we found fewest recurrences in cALN < 4 patients with MARI-node pCR and most recurrences in patients staged cALN ≥ 4, ypMARI-pos who underwent ALND plus ART. Baseline factors associated with disease recurrence in multivariable analysis were residual tumor in the MARI-node (HR 3.1) and triple-negative subtype (HR 4.3).

Post-NST axillary staging strategies for cN+ patients other than the MARI-procedure include the post-NST sentinel lymph node biopsy (SLNB) and targeted axillary dissection (TAD) [8], which combines removal of a pre-NST clipped node with SLNB [4, 5]. The accuracy of the post-NST SLNB is a much-debated topic. While the MARI-procedure has a false-negative rate (FNR) of 7% with a risk of undertreatment in only 3% of patients [10, 11], FNRs of 8% to 40% have been reported for the post-NST SLNB [5, 7, 32, 33]. A clinically considered acceptable FNR of ≤ 10% was only achieved when three or more sentinel nodes (SNs) were removed and dual-tracer mapping was used [7, 33]. In the ACOSOG Z1071 and SENTINA trial, retrieval of three or more SNs occurred only in 56% and 34% of patients, respectively [7, 33].

The FNR of TAD was reported to be as low as 2–4% [8–10, 34], and could be lower than the FNR of the MARI-procedure due to assessment of more ALNs. In the study by Caudle et al. [8], three or more ALNs were removed in 47% (63 of 134) of patients, while a median of only one (IQR 1–2) ALN is removed with the MARI-procedure. Compared to the MARI-procedure, TAD also requires an additional visit to the outpatient clinic for both the localization of the clipped node and the sentinel-node procedure.

Although the removal of more ALNs may decrease the FNR, it also increases the risk of lymphedema [35]. Moreover, it is important to note that lowering the FNR of post-NST axillary staging methods further below 10% may not significantly lower the axillary recurrence rate. With the MARI-procedure, we found an excellent three-year aRFI of 98%.

Several other studies indicate that limited axillary residual disease may safely be left in situ without compromising aRFI. In patients treated with SLNB in the primary surgery setting, 5–10 year axillary recurrence rates of 0–2% were found, which is lower than expected based on the reported FNRs of 5–10% [4, 20, 36–40], and the ACOSOG Z0011 and IBCSG 23–01 trials reported excellent locoregional control in patients with limited disease at SLNB without further axillary treatment [20, 36]. In addition, the AMAROS trial found that ART was as effective as ALND for the treatment patient with tumor-positive SLN’s (5-year axillary recurrence of 1.2% vs. 0.4%) [41]. Of note, four or more tumor-positive ALNs (pN2) were found in 8% of the patients in the ALND-arm, which supports the efficacy of ART even in patients with higher axillary tumor load.

Reports on axillary recurrence after de-escalated locoregional axillary treatment in cN+ patients with NST are limited. Four- and five year recurrence rates of 2% and 0% were described in cN1 patients with a tumor-negative post-NST SLNB in whom ALND was omitted [38, 42, 43]. Results of comprehensive trials investigating the impact of de-escalated axillary treatment after NST such as the ongoing NSABP B-51/RTOG 1304 (NCT01872975) [44] and the Alliance A011202 trial (NCT01901094) [45], are currently unknown. In addition, whether ALND can be avoided after NST in patients with cN2-3 disease is not investigated in these trials [46]. Notably, in the present study we showed that the MARI-protocol is not only an effective method for de-escalation of axillary treatment in cN1 patients, but also for patients with more extensive axillary disease prior to NST.

Limitations to implementation of the MARI-protocol could be the use of radioactive iodine seeds. Although iodine seeds are increasingly being used for tumor localization due to improved surgical planning and diminished patient discomfort [16], extensive regulations often apply for handling and disposal of the seeds. According to our protocol, iodine seeds should be allowed to remain in situ for the duration of NST.

Furthermore, FDG-PET/CT it is not yet part of the diagnostic work-up for cN+ breast cancer patients in several countries. The costs (± €1260[47] [$1545[48]]) may therefore not always be fully covered by health insurance [47–49]. Staging breast cancer patients with FDG-PET/CT, however, can replace diagnostic imaging with CT, chest X-ray and ultrasound with higher diagnostic accuracy and cost-effectiveness [50, 51]. In addition, the diagnostic accuracy of FDG-PET/CT for axillary staging is higher compared to other modalities and therefore essential when tailoring axillary treatment [52–54].

Limitations of this study are its single-center character and prospective registration design. Ten percent of the patients undergoing tailored axillary treatment after NST according to the MARI-protocol were excluded from analysis due to deviations from the protocol. The type of protocol violations varied, and included both patients with overtreatment (e.g. cALN ≤ 4 patients with intraoperatively assessed extensive residual axillary disease treated with ALND) as well as patient who were undertreated (no ALND or ART in case of a tumor-positive MARI-node) according to protocol.

In conclusion, in this study we demonstrated that the MARI-protocol is an effective axillary staging and treatment algorithm which resulted in omission of ALND in 80% of cN+ patients undergoing NST while maintaining excellent three-year axillary- and regional RFI rates of 98% and 96%. Therefore, the MARI-protocol may be considered a suitable method to de-escalate axillary treatment in selected patients. Longer follow-up is needed to evaluate these results at five- and ten years follow-up.

## Declarations

### Availability of data and material

The datasets generated for the current study are available from the corresponding author on reasonable request.
